# Prevention of Lower Limb Muscle Atrophy With Neuromuscular Electrical Stimulation in a Patient With Tachycardia-Induced Cardiomyopathy Secondary to Thyroid Storm: A Case Report

**DOI:** 10.7759/cureus.101732

**Published:** 2026-01-17

**Authors:** Shinya Sato, Kei Imaoka, Shuri Nakao, Norimasa Egusa, Sokichi Maniwa

**Affiliations:** 1 Division of Rehabilitation Medicine, Shimane University Hospital, Izumo, JPN; 2 Department of Rehabilitation Medicine, Shimane University Faculty of Medicine, Izumo, JPN

**Keywords:** cardiac rehabilitation (cr), neuromuscular electrical stimulation, quadriceps muscle thickness, tachycardia-induced cardiomyopathy, thyroid storm

## Abstract

Tachycardia-induced cardiomyopathy (TIC) is reversible; however, the acute phase is often accompanied by hemodynamic instability, which makes conventional exercise therapy difficult to implement. Neuromuscular electrical stimulation (NMES) has been suggested as a potential alternative strategy to prevent muscle atrophy. Herein, we report a case in which NMES was used in a patient during the acute phase of TIC, secondary to thyroid storm, highlighting its safety and potential effectiveness. A male in his 40s was diagnosed with TIC associated with thyroid storm. On admission, the patient presented with atrial fibrillation, severe cardiac dysfunction with a left ventricular ejection fraction of 19%, and markedly elevated thyroid hormone levels. Due to unstable hemodynamics, conventional exercise therapy was deferred, and NMES was initiated on day nine of hospitalization, and it was continued until day 12. It was performed once daily for 20 minutes. Muscle ultrasonography revealed that quadriceps muscle thickness had decreased by approximately 5% during hospitalization. Importantly, no hemodynamic deterioration or adverse events were observed. From day 13, aerobic exercises using a cycle ergometer and resistance training were initiated. The patient achieved a six-minute walk distance of 545 m and was discharged on day 28. NMES was safely implemented without adverse events, even during periods of hemodynamic instability in a patient with TIC. In situations where conventional exercise therapy is limited due to hemodynamic instability, NMES may represent a feasible intervention option. The findings of this single case are hypothesis-generating and warrant further investigation.

## Introduction

Tachycardia-induced cardiomyopathy (TIC) is a reversible form of cardiomyopathy characterized by left ventricular dysfunction secondary to persistent tachyarrhythmia, which may occur in association with hyperthyroidism and related conditions [1]. Previous studies have shown that improvement in tachycardia generally requires approximately one to two weeks, whereas left ventricular ejection fraction (LVEF) and cardiac output may continue to deteriorate for up to four weeks [2]. Furthermore, even after heart rate control is achieved, diastolic and systolic dysfunction of the left ventricle may persist for approximately one week [3]. Consequently, the management of TIC requires a prolonged treatment period, during which bed rest often leads to physical deconditioning. Therefore, physiotherapy aimed at preventing functional decline is essential, even during the early phase when there is hemodynamic instability.

However, according to the current guidelines, intensive exercise therapy is regarded as a relative contraindication in patients with uncontrolled tachyarrhythmia [4], and conventional rehabilitation is frequently difficult to implement.

Neuromuscular electrical stimulation (NMES) has been reported to be a safe and feasible alternative to active exercise in critically ill patients and in those with acute heart failure, without adverse events [5,6]. Accordingly, NMES may also be a useful option in the acute phase of TIC when hemodynamic instability precludes conventional exercise therapy. Nevertheless, clinical evidence regarding the use of NMES during the acute phase of TIC remains extremely limited. Herein, the acute phase of TIC is defined as the period during which persistent tachyarrhythmia and hemodynamic instability are present, prior to clinical stabilization. We report a case in which NMES was safely introduced during this phase.

## Case presentation

The patient was a male in his 40s, with a height of 173.0 cm, body weight of 58.2 kg, and a body mass index of 18.2 kg/m². The patient was diagnosed with thyroid storm and TIC. Prior to admission, he was independent in activities of daily living and employed in sales, which was estimated to require approximately 4.0 metabolic equivalents (METs). His medical history was notable for hyperthyroidism, which had been diagnosed two years earlier, and oral medication was initiated at that time. However, he discontinued the medication by his own volition, approximately six months before admission. A few days before admission, the patient had palpitations, exertional dyspnea, and gastrointestinal symptoms, including diarrhea, and consulted a local physician. He was found to have tachyarrhythmic atrial fibrillation, impaired cardiac function, and markedly elevated thyroid hormone levels, and he was subsequently diagnosed with congestive heart failure due to thyroid storm and TIC. He was transferred to our hospital via emergency transport and admitted on the same day. Thyroid storm was diagnosed based on the diagnostic criteria of the Japan Thyroid Association [7]. Written informed consent was obtained from the patient for the publication of this case report after he was provided with an explanation in accordance with the Declaration of Helsinki.

Hospital course upon admission

**Figure 1:**
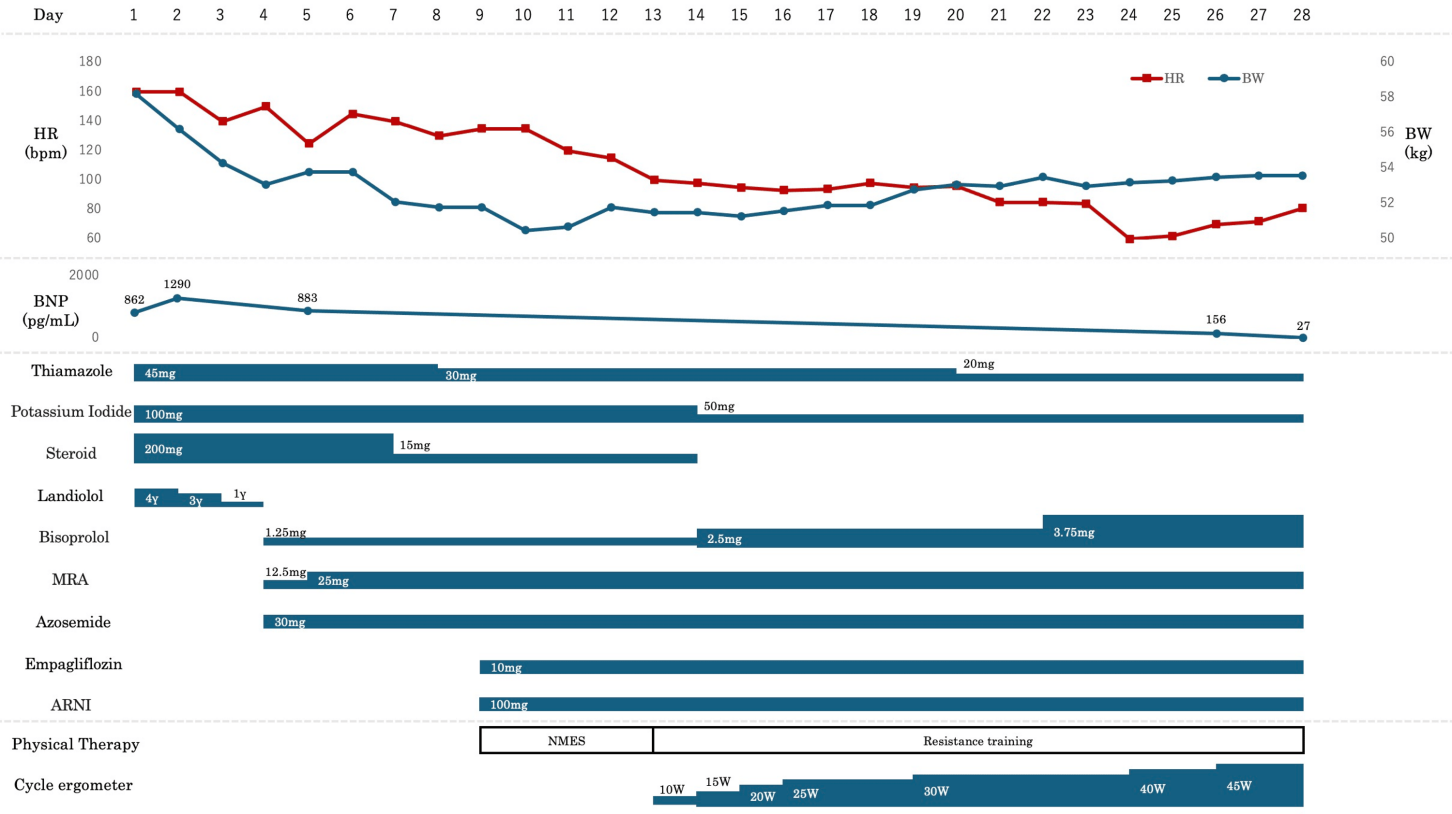
Clinical course. HR, heart rate; BW, body weight; BNP, brain natriuretic peptide; MRA, mineralocorticoid receptor antagonist; ARNI, angiotensin receptor neprilysin inhibitor; NMES, neuromuscular electrical stimulation.

At admission, the patient’s blood pressure was 141/94 mmHg, and heart rate was 167 bpm. Transthoracic echocardiography revealed an LVEF of 19%, moderate mitral regurgitation, and diffuse hypokinesis of the left ventricle. Laboratory tests revealed a brain natriuretic peptide (BNP) level of 883 pg/mL, a free tri-iodothyronine (FT3) level >25 pg/mL, and a free thyroxine (FT4) level >8.0 ng/dL, consistent with thyrotoxicosis. Pharmacological treatment for thyroid storm was initiated with thiamazole, potassium iodide, and hydrocortisone sodium succinate (200 mg). In parallel, therapy for TIC included landiolol. Following these interventions, the hemodynamic status gradually stabilized, and the FT3, FT4, and BNP levels decreased. Body weight also decreased from 58.2 kg at admission. The clinical course after admission is summarized in Figure 1.

Physical therapy course

Physical therapy was initiated on day eight of hospitalization. At that time, blood pressure was 128/54 mmHg, heart rate was 130 beats/min, and SpO2 was 98% on room air. Electrocardiography revealed atrial fibrillation. There was no lower leg edema. At the time of intervention, nutritional status was assessed using the Controlling Nutritional Status (CONUT) score, which was 5. Standing and walking were independent. However, because tachyarrhythmic atrial fibrillation was present, even at rest, the heart rate increased to 150 beats/min during bed-based muscle training or short-distance ambulation. After discussion with the attending physician, active exercise therapy, such as lower-limb strength training, was withheld.

Due to concerns regarding lower limb muscle weakness associated with prolonged bed rest, NMES was initiated on day nine to prevent muscle atrophy. NMES was performed using a low-frequency device (AUTO TENS-PRO®, Homer Ion Laboratory, Tokyo, Japan), in accordance with the protocol described by Tanaka et al. [8]. Belt electrodes were applied bilaterally to the proximal and distal quadriceps and to the distal lower legs, thereby stimulating the thigh and more distal lower-limb muscles. The stimulation parameters were set at a frequency of 20 Hz, pulse width of 250 μs, and duty cycle of 5:2 (5 s contraction/2 s relaxation), with the intensity adjusted to the maximum tolerable level. NMES was performed once daily for 20 minutes. For safety management, blood pressure and heart rate were measured before and after each session, and continuous electrocardiographic monitoring was performed during stimulation. Discontinuation criteria, based on guidelines [4] and the report by Iwatsu et al. [9], included a change in systolic blood pressure >20 mmHg, a change in heart rate >20 beats/min, or the presence of life-threatening arrhythmias.

The effectiveness of NMES was evaluated using quadriceps muscle thickness, which was assessed using ultrasonography. Ultrasound measurement of muscle thickness is a noninvasive and easily applicable bedside technique, and its reliability and validity have been reported even in patients with heart failure [10]. Moreover, quadriceps muscle thickness has been shown to correlate with muscle volume [11]. A portable ultrasonography device (miruco®, Nippon Sigmax Corporation, Tokyo, Japan) was used with a linear probe in B-mode. Measurements were performed with the patient in the supine position, hips in neutral alignment, and knees extended. The measurement site was defined as the midpoint between the anterior superior iliac spine and the upper border of the patella [12]. All ultrasound measurements of muscle thickness were performed by a single assessor.

Quadriceps muscle thicknesses prior to NMES (day eight) were 21 mm (right) and 20 mm (left). Vital signs immediately before the initiation of NMES were as follows: blood pressure of 128/54 mmHg, heart rate of 130 beats/min (atrial fibrillation), and SpO₂ of 98%. At the end of the session, blood pressure was 125/54 mmHg, heart rate was 130 beats/min (atrial fibrillation), and SpO₂ was 98%. No symptoms of discomfort, worsening of heart failure, or other adverse events were observed during or after the NMES sessions. After four consecutive days of NMES (day 12), the thicknesses were 20 mm (right) and 20 mm (left). No vital sign instabilities or adverse events were observed during NMES, and all sessions were conducted using the same predefined stimulation settings without interruption or modification.

Following improvement in tachycardia, the patient transitioned to aerobic exercises using a cycle ergometer and lower-limb resistance training on day 13. Exercises were performed with a Strength Ergo® device (Fukuda Denshi, Tokyo, Japan), starting at 0 W and gradually increasing to 45 W, depending on symptoms. There were no adverse events during the exercises.

On day 26, sinus rhythm was restored by catheter ablation. By day 27, LVEF had improved to 41%. Functional assessments at that time revealed a six-minute walk distance of 545 m, a Short Physical Performance Battery score of 12, and a peak oxygen uptake (peak VO₂) of 18.8 mL/min/kg (61% of predicted; 5.4 METs). Also, the quadriceps muscle thicknesses were 20 mm (right) and 19 mm (left). The patient was discharged on day 28. The clinical course of physical function at the initiation of physical therapy, at the initiation of aerobic exercise therapy, and at discharge is summarized in Table 1.

**Table 1:** Cardiac function, physical function, and exercise tolerance at the initiation of physical therapy, initiation of aerobic exercise, and at discharge. LVEF, left ventricular ejection fraction; LVDd, left ventricular end-diastolic dimension; LVDs, left ventricular end-systolic dimension; SPPB, Short Physical Performance Battery; 6MWD, six-minute walk distance; VO₂, oxygen uptake; VE/VCO₂, minute ventilation/carbon dioxide production slope; N/A, not available.

Variable	Initiation of physical therapy	Initiation of aerobic exercise	At discharge
LVEF (%)	19	28	41
LVDd/LVDs (mm)	58/50	42/36	54/40
Grip strength (kg: right/left)	21.3/20.5	20.8/20.7	22.5/21.5
Quadriceps muscle thickness (mm: right/left)	21/20	20/20	20/19
Knee extensor muscle strength (kgf/kg: right/left)	N/A	N/A	0.47/0.37
SPPB (points)	N/A	12	12
6MWD (m)	N/A	N/A	545
Peak VO_2 _(ml/min/kg)	N/A	N/A	18.8
Peak VO_2_ (ml/beats）	N/A	N/A	10.8
Minimum VE/VCO_2_	N/A	N/A	21.1

## Discussion

This case involved the introduction of NMES as an alternative to conventional exercise therapy for a patient during the acute phase of TIC secondary to thyroid storm. To the best of our knowledge, this is the first report of a physiotherapy intervention during the acute phase of TIC.

The patient presented with tachyarrhythmic atrial fibrillation and severe left ventricular dysfunction upon admission, making aggressive exercise therapy difficult to perform. Nevertheless, NMES was safely administered without worsening hemodynamic status. Previous studies have reported the feasibility and safety of NMES in acute heart failure [5]. In contrast, TIC is characterized by persistent tachyarrhythmia, which may limit the safety of exercise therapy. Therefore, this case has clinical significance in extending the applicability of NMES to a more unstable physiological context beyond what has been documented in prior studies.

Furthermore, NMES appeared to suppress muscle atrophy. Previous studies have reported that improvement in tachycardia requires a prolonged period [2], and during this treatment period, progression of lower limb muscle atrophy due to rest is a concern. In this case, NMES was initiated on day nine, the day after initiation of physical therapy, and continued for four days until day 12. The results showed that the quadriceps thickness decreased only minimally, from 21 mm (right) and 20 mm (left) at baseline to 20 mm and 19 mm at the completion of NMES and remained at 20 mm and 19 mm at discharge, representing a reduction of approximately 5% throughout hospitalization. However, the minimal clinically important difference for ultrasound-measured quadriceps thickness has not been established, and this change should be interpreted descriptively and with caution. Matsuo et al. [13] reported that quadriceps thickness decreased by 13.5% within two weeks of hospitalization for patients with acute heart failure and that NMES suppressed muscle atrophy in this cohort. Similarly, studies on critically ill patients have also demonstrated suppression of muscle wasting with NMES [14]. Taken together, these findings suggest that NMES may have contributed to attenuating the progression of muscle atrophy in this patient. Previous studies [13,15] have introduced NMES earlier and applied it for a longer duration. In contrast, the intervention in this report was initiated later and applied for a shorter period, primarily as an alternative to resistance training. Nonetheless, Iwatsu et al. [9] demonstrated that even short-term NMES suppressed continuous protein degradation and preserved muscle function, which is consistent with our findings. Furthermore, in this case, corticosteroids were administered as part of the treatment for thyroid storm, and glucocorticoids have been reported to suppress muscle protein synthesis and promote muscle atrophy [16,17]. Accordingly, the patient was considered to be at substantial risk of muscle wasting due to physical inactivity and steroid therapy. In contrast, NMES has been reported to stimulate muscle protein synthesis and reduce proteolytic activity, suggesting a potential counteracting mechanism against glucocorticoid-induced muscle catabolism [18]. Therefore, despite these catabolic risk factors, NMES may have attenuated the progression of muscle loss in this case. Quadriceps muscle thickness has been reported to be an important prognostic factor for patients with heart failure, particularly for older adults [10]. Therefore, the present report highlights the clinical significance of introducing NMES as early as possible, even in hemodynamically unstable patients who cannot undergo conventional exercise therapy. In this patient, transition to aerobic exercise and resistance training was feasible after NMES, ultimately resulting in improved exercise tolerance, including a peak VO₂ of 18.8 mL/min/kg and a six-minute walk distance of 545 m, which enabled discharge. In contrast, peak VO₂ at discharge remained at 61% of the predicted value, indicating reduced exercise tolerance. Cardiac function was still recovering at the time of discharge. In addition, hyperthyroidism, which was the underlying disease in this patient, has been reported to accelerate catabolism of muscle protein through excessive secretion of thyroid hormones, leading to muscle weakness and reduced mitochondrial efficiency [19]. In the present patient, skeletal muscle dysfunction was also evident, including reduced knee extensor strength relative to body weight. In addition, aerobic exercise is considered to be more effective than NMES for improving peak VO₂ [20]. Therefore, the relatively short duration of aerobic exercise after NMES (approximately two weeks) may have contributed to the persistently low peak VO₂ observed at discharge.

This report has several limitations. First, data on quadriceps muscle thickness from the early phase of hospitalization were not available, making it impossible to accurately assess changes prior to the introduction of NMES. Second, because this report describes a single case without a control group, causal inferences cannot be drawn, and both the interpretation and generalizability of the findings should be approached with caution. In addition, the limited degree of muscle loss observed in this case cannot be attributed solely to NMES and may have been influenced by confounding factors, including the patient’s young age and independence in standing and ambulation. Furthermore, restoration of sinus rhythm by catheter ablation prior to discharge may have influenced exercise tolerance outcomes, and its effects cannot be separated from those of NMES or other rehabilitation interventions. Nevertheless, this report suggests that NMES can be safely implemented even during the acute phase of TIC and provides a basis for future studies exploring appropriate timing and patient selection for NMES initiation.

## Conclusions

NMES was safely implemented without adverse events, even during periods of hemodynamic instability in a patient with TIC. In situations where conventional exercise therapy is limited due to hemodynamic instability, NMES may represent a feasible intervention option. The findings of this single case are hypothesis-generating and warrant further investigation.
